# 4304 Immune markers in tumor immune microenvironment of neuroblastoma correlate with risk groups

**DOI:** 10.1017/cts.2020.403

**Published:** 2020-07-29

**Authors:** Robyn Denise Gartrell, Hanna M. Moisander-Joyce, Andrew M. Silverman, Helen E. Remotti, Darrell J Yamashiro

**Affiliations:** 1 Columbia University, Irving Institute for Clinical and Translational Research; 2 Columbia University Irving Medical Center; 3 Hackensack Medical Center

## Abstract

OBJECTIVES/GOALS: Neuroblastoma (NB) is the most common extra-cranial solid tumor with outcomes varying from spontaneous regression to metastatic with high mortality rates. The tumor immune microenvironment (TIME) may play a significant role in this disease. In this study we analyze the TIME comparing high-risk (HR) and low-risk (LR) NBs using multiplex platforms. METHODS/STUDY POPULATION: Two tissue microarrays (TMAs) with 2mm cores were created from 41 patients treated at Columbia University Irving Medical Center. Five micron TMA slides were stained for Digital Spatial Profiling (DSP, nanoString) and multiplex immunofluorescence (mIF). For DSP, a 24-patient subset including 11 HR, 8 LR and 4 intermediate risk patients was analyzed for 34 proteins. Protein expression among risk groups was compared using Mann-Whitney t-test. For mIF, TMA FFPE slides were stained for DAPI, CD3, CD8, CD68, HLA-DR, PDL1 and Chromogranin A. Whole TMA cores were captured as 9 -20X multispectral images (MSIs) stitched into a 3x3 MSI using Vectra (Akoya). MSIs were processed with inForm and qualitative analysis performed comparing HR and LR tumors. RESULTS/ANTICIPATED RESULTS: With DSP, we find significantly more HLA-DR in HR compared to LR tumors (p = 0.016). When controlling for immune cells with CD45 we find HLA-DR/CD45 to be higher in HR than LR tumors (p = 0.026). We found increased PD1 and PDL1 expression in all groups without significant difference between LR and HR (p = 0.778 and p = 0.310, respectively). Preliminary analysis of mIF on 9 patients (4 HR and 5 LR) finds HR tumors appear to have more immune cells than LR tumors, specifically more CD3+CD8- T cells while total CD8+ cells may be similar. There may be less macrophages in the HR compared to LR tumors. Completion of image processing and quantitative analysis of mIF data is underway. DISCUSSION/SIGNIFICANCE OF IMPACT: Increased expression of immune markers in NB TIME correlates with higher risk, which is unlike many other tumors. We compared TIME in HR and LR NB using multiplex platforms, DSP and mIF. We find that HLA-DR is more expressed in HR NB while PD1 and PDL1 expression is consistently high and not different between risk groups. Further analysis is underway. CONFLICT OF INTEREST DESCRIPTION: Robyn D. Gartrell-Corrado received grant support from nanoString for Digital Spatial Profiling and received honoraria and travel support from Northwest Biotherapeutics and PerkinElmer, respectively.

